# A Short Way with Anti-Vaccinators

**Published:** 1898-01-22

**Authors:** 


### EDITOR'S LETTER-BOX.

[Our Correspondents are reminded that prolixity is a great bar to publication, and that brevity of style and conciseness of statement greatly facilitate early insertion.]

### A SHORT WAY WITH ANTI-VACCINATORS.

DR. FRANCIS BOND, hon. secretary Jenner Society, writes: I shall be glad if you will allow me at this early period of the New Year to invite the attention of your readers to the need for their co-operation in meeting the very active agitation which is being carried on by the Anti-Vaccination League at the present time. In view of the probability of legislation during the ensuing session of Parliament, in furtherance of the recommendations of the late Royal Commission, an effort is being made to influence the members of the Legislature, both directly, by personal communications, and indirectly, through their local constituents. To effect this latter object letters are addressed to such local publications as will admit them, not only attacking compulsory vaccination, but seeking to discredit vaccination itself. It is of the greatest importance that these letters, wherever they appear, should be answered It has been too much the practice hitherto to disregard them as in most cases unworthy of notice. To this mistaken policy is in a large degree attributable the hold the anti-vaccination agitation has obtained in many localities. To reply to them is useful in two ways; it serves at the same time to neutralise misrepresentations and to educate the public to appreciate the facts on which vaccination rests. This is work which must be done mainly by the medical profession, for few others are in a position to do it. But there is another reason why it is incumbent on the profession to undertake it. Anti-vaccinators constantly make it a subject of complaint that vaccination is the only medical prescription which is enforced by law. This is true; and it is, therefore, only right that as the maintenance of vaccination so largely rests upon medical opinion every effort should be made to instruct the public as to the justification for this position. I trust, therefore, that whenever any of your readers see a statement in any local paper, whether in the form of a paragraph or letter, misrepresenting the case for vaccination, if they do not themselves reply to it, they will send the paper to the Jenner Society, that it may be dealt with if practicable and desirable. I have, on behalf of the society during the past year, replied to a large number of such letters, but it is only possible for me to do a limited amount of work of this kind, and it is much better that local attacks should, if possible, be met by local defence. I shall at all times be glad to give any assistance towards this work. It is a form of warfare which needs some little training to ensure success, for the enemy is crafty, is familiar with the use of his weapons, and too often does not mind striking a foul blow when a fair one fails.

